# Sinonasal Sarcomas Management: An International Consensus Statement

**DOI:** 10.1002/alr.70038

**Published:** 2025-09-30

**Authors:** Alessandro Vinciguerra, Francesca Caspani, Marco Valentini, Anna Maria Camarda, Salvatore Provenzano, Eric W. Wang, Cem Meço, Marco Ferrari, Pavol Surda, Semi Harrabi, Fernando Augusto Batista Campos, Javier Martin Broto, B. Ashleigh Guadagnolo, Pierina Navarria, Ali Hosni, Nadia Hindi, Antoine Italiano, Daniel M. Trifiletti, Bernd Kasper, Benjamin Verillaud, Alberto Schreiber, Paolo Castelnuovo, Piero Nicolai, Iacopo Dallan, Matias Chacon, Shirley Y. Su, Juliette Thariat, Claudia Valverde, Matt Lechner, Jean Yves Blay, Maria Rosaria Fiore, Jean Anderson Eloy, Mehdi Brahmi, James N. Palmer, Victor Castro Oliden, Daniela Greto, Iwona Lugowska, Angelo Paolo Dei Tos, Thibaut van Zele, Alkis J. Psaltis, Paolo Giovanni Casali, Armelle Dufresne, Claudia Sangalli, Christos Georgalas, Ester Orlandi, Peter‐John Wormald, Philippe Herman, Ricardo Carrau, Ehab Y. Hanna, Lisa Licitra, Mario Turri‐Zanoni, Paolo Battaglia

**Affiliations:** ^1^ Division of Otorhinolaryngology Department of Biotechnology and Life Sciences University of Insubria ASST Lariana Como Italy; ^2^ Head and Neck Medical Oncology Department ASST Lariana Como Italy; ^3^ Radiation Oncology Clinical Department National Center for Oncological Hadrontherapy (CNAO) Pavia Italy; ^4^ Department of Clinical Surgical, Diagnostic, and Pediatric Sciences University of Pavia Pavia Italy; ^5^ Department of Oncology and Hematology Fondazione IRCCS Istituto Nazionale Dei Tumori Milan Italy; ^6^ Department of Otolaryngology University of Pittsburgh Medical Center Pittsburgh Pennsylvania USA; ^7^ Department of Otorhinolaryngology – Head and Neck Surgery Ankara University Medical School Ankara Turkiye; ^8^ Department of Otorhinolaryngology ‐ Head and Neck Surgery Salzburg Paracelsus Medical University Salzburg Austria; ^9^ Department of Otolaryngology ‐ Head and Neck Surgery Cornell University Weill Cornell Medical College New York New York USA; ^10^ Section of Otorhinolaryngology – Head and Neck Surgery Department of Neuroscience (DNS) University of Padova Padova Italy; ^11^ Unit of Otorhinolaryngology – Head and Neck Surgery “Azienda Ospedale‐Università Padova” Padova Italy; ^12^ Otolaryngology Guy's and St Thomas' Hospitals NHS Trust London UK; ^13^ Heidelberg Ion‐Beam Therapy Center (HIT) Department of Radiation Oncology Heidelberg University Hospital Heidelberg Germany; ^14^ Department of Medical Oncology A.C.Camargo Cancer Center São Paulo Brazil; ^15^ Medical Oncology Department Fundacion Jimenez Diaz University Hospital University Hospital General De Villalba Madrid Spain; ^16^ Instituto De Investigacion Sanitaria Fundacion Jimenez Diaz (IIS/FJD; UAM) Madrid Spain; ^17^ Department of Radiation Oncology University of Texas MD Anderson Cancer Center Houston Texas USA; ^18^ Department of Radiotherapy and Radiosurgery IRCCS Humanitas Research Hospital Rozzano Milan Italy; ^19^ Radiation Medicine Program, Princess Margaret Cancer Centre University Health Network University of Toronto Toronto Ontario Canada; ^20^ Faculty of Medicine University of Bordeaux Bordeaux France; ^21^ Department of Radiation Oncology Mayo Clinic Jacksonville Florida USA; ^22^ Sarcoma Unit, University of Heidelberg Mannheim Cancer Center (MCC) Mannheim University Medical Center Mannheim Germany; ^23^ Otorhinolaryngology and Skull Base Center AP‐HP, Hôpital Lariboisière Université De Paris Paris France; ^24^ Unit of Otorhinolaryngology Manerbio and Desenzano Hospitals ASST Del Garda Brescia Italy; ^25^ Skull Base and Rhino‐orbital Surgery Unit Azienda Ospedaliero‐Universitaria Pisana Pisa Italy; ^26^ Department of Medical Oncology Alexander Fleming Cancer Institute Buenos Aires Argentina; ^27^ Department of Head and Neck Surgery The University of Texas MD Anderson Cancer Center Houston Texas USA; ^28^ Department of Radiation Oncology Centre François‐Baclesse Caen France; ^29^ Université De Normandie Caen France; ^30^ Department of Medical Oncology Vall D'hebron University Hospital Barcelona Spain; ^31^ Division of Surgery and Interventional Science and UCL Cancer Institute London UK; ^32^ Medical Oncology Department Centre Leon Berard Lyon France; ^33^ Department of Otolaryngology Head and Neck Surgery Rutgers New Jersey Medical School Newark New Jersey USA; ^34^ Department of Otorhinolaryngology Head and Neck Surgery Perelman School of Medicine University of Pennsylvania Philadelphia Pennsylvania USA; ^35^ Department of Oncology Instituto Nacional de Enfermedades Neoplásicas Lima Peru; ^36^ Azienda Ospedaliero‐Universitaria Careggi Radiation Oncology Unit Florence Italy; ^37^ Maria Sklodowska‐Curie Institute of Oncology Warsaw Warszawa Poland; ^38^ Surgical Pathology and Cytopathology Unit Department of Medicine‐ DIMED University of Padua School of Medicine Padua Italy; ^39^ Department of Otorhinolaryngology Ghent University Hospital Ghent Belgium; ^40^ Department of Otolaryngology, Head and Neck Surgery Queen Elizabeth Hospital Adelaide South Australia Australia; ^41^ Department of Radiation Therapy Fondazione IRCCS Istituto Nazionale Dei Tumori Milan Italy; ^42^ Department of Otorhinolaryngology, Head and Neck Surgery University of Nicosia Medical School Nicosia Cyprus; ^43^ Department of Otolaryngology, Head and Neck Surgery The Ohio State University Columbus Ohio USA

**Keywords:** angiosarcoma, biphenotypic sinonasal sarcoma, chondrosarcoma, Ewing sarcoma, leiomyosarcoma, malignant peripheral nerve sheath tumor, osteosarcoma, rhabdomyosarcoma, sinonasal sarcomas, synovial sarcoma

## Abstract

**Introduction:**

Sinonasal sarcomas are exceedingly rare entities, constituting less than 7% of head and neck sarcomas. Their complex histology needs specialized treatment, which is often based on multimodal approaches including surgery, radiation therapy, and/or chemotherapy. This manuscript aims to gather expert opinions to establish common management principles for sinonasal sarcomas.

**Methods:**

This international consensus followed a modified Delphi method in seven steps, including statements definition by the core group, expert panel recruitment, and a two‐round survey. Sixty‐two statements on sinonasal sarcoma management were developed. Experts from multiple continents participated, and results were anonymized and analyzed between March and May 2025.

**Results:**

A total of 44 invited experts were recruited, 43.2% otorhinolaryngologists/head and neck surgeons, 31.8% medical oncologists, and 25% radiation oncologists. Participants varied in age and experience, representing Europe (70.5%), North America (18.2%), South America (6.8%), and Asia (4.5%). Among all histologies, biphenotypic sarcoma, chondrosarcoma, leiomyosarcoma, and myofibrosarcoma are principally treated with an upfront surgical management, differently from Ewing sarcoma and rhabdomyosarcoma in which chemotherapy, eventually associated with radiotherapy, is often chosen. In the remaining histologies (angiosarcoma, liposarcoma, malignant peripheral nerve sheath tumor [MPNST], osteosarcoma, and synovial sarcoma), a precise multimodal treatment is less standardized and needs to be discussed on a case‐by‐case basis.

**Conclusion:**

Sinonasal sarcomas require a histology‐driven approach to determine upfront treatment, whether surgical, medical, or multimodal. Despite this structured strategy, prognosis remains highly variable across subtypes. Multidisciplinary evaluation and individualized management in referral centers are crucial to address the biological diversity and anatomical complexity of these rare malignancies.

## 1 Introduction

Sarcomas of the sinonasal cavities are an extremely rare condition that accounts for less than 7% of all head and neck sarcomas, an entity that comprises less than 1% of head and neck malignancies [1]. In addition to being rare, sarcoma histologies of the sinonasal tract are extremely complex and need expert immunohistochemical and genetic analysis to distinguish the different subtypes that, however, may still present overlaps, especially with variations in tumor grade or dedifferentiation [1, 2]. As a result, for many authors, the pathological diagnosis of these entities should be centralized in experienced tertiary referral centers [3].

Due to their rarity and complex diagnosis, in the literature, the management of sinonasal sarcomas (SNS) is, on one hand, based on limited case series, and, on the other, generally pooled together with all head and neck sarcomas, thus limiting the ability to distinguish management nuances. Nevertheless, compared to sarcomas of the trunk and extremities, SNS are known to be more challenging to treat due to their proximity to the orbit and skull base, which does not allow wide surgical excision of these pathologies [4, 5]. Indeed, even if surgery represents the cornerstone of treatment for the majority of SNS, achieving healthy margins of resection for these entities is generally challenging. Specifically, considering the wider experience of sarcomas of the extremities, the current surgical standard of treatment for SNS is represented by healthy margins (e.g. >4 cm), which is hardly ever achievable in the sinonasal compartment [6] and justifies one of the worst prognoses among sarcoma localizations [4].

For these reasons, SNS management is generally based on a multimodal therapy, a treatment modality that includes surgery and/or radiation therapy for local control and chemotherapy (CHT) for systemic control. These therapeutic options are thought to be personalized for each histology and disease extent. Unfortunately, due to their rarity, no solid treatment evidence is available for each SNS subtype, and their management is generally based on each center's experience [2].

This manuscript aims to collect opinions of experts through an international consensus statement (Delphi consensus), aiming to pool together experiences of tertiary referral centers of these entities, producing common principles of management for SNS.

## 2 Methods

This international consensus statement consisted of seven steps, following the Delphi method [7]: (1) Determination of scope and population of interest, (2) statements definition by the core group, (3) experts panel recruitment, underlying potential conflict of interest, (4) qualitative survey and development of an initial set of statements with open feedback from panelist, (5) revision of ambiguous survey questions and adaptation of statements, (6) second round of Delphi survey, (7) data analysis and publication.

Specifically, the scientific committee initially elected a core group, internationally recognized experts (three otorhinolaryngologists—head and neck surgeons, two radiation oncologists, and two medical oncologists), and an expert group of 44 panelists, including experts selected based on bibliometrics and clinical expertise in the management of SNS.

The project was planned between September 2024 and February 2025 and presented for consensus between March and May 2025.

Initially, the core group members were asked to formulate statements based on the available literature on the following topics: In (1) general principles and open issues concerning SNS (2) management guidelines concerning each SNS histology based on WHO fifth edition: (A) angiosarcoma, (B) biphenotypic sinonasal sarcoma, (C) chondrosarcoma, (D) Ewing sarcoma, (E) leiomyosarcoma, (F) liposarcoma, (G) low grade myofibroblastic sarcoma (myofibrosarcoma), (H) malignant peripheral nerves sheath tumor (MPNST), (I) osteosarcoma, (L) rhabdomyosarcoma, and (M) synovial sarcoma. For each topic, the core group had to define the specific management modalities based on pathologic status and patient conditions and had to provide the relevant literature and level of evidence (according to the Oxford level of evidence) [7, 8]. Afterwards, the scientific committee reviewed the list of statements to eliminate duplicates, integrate missing fields, and provide a unified level of evidence for each SNS treatment modality.

A total of 62 statements were produced (Supporting Information 1); the final list of statements was voted upon using a modified Delphi method [7]. A Likert‐type scale with 7 points was used to rate treatment options, as follows: 0 = I don't know, 1 = strongly disagree; 2 = disagree; 3 = somewhat disagree; 4 = neither agree nor disagree; 5 = somewhat agree; 6 = agree; and 7 = strongly agree.

Consensus was achieved when the following two criteria were met:

- 80% of votes fell within the 2 upper categories (6 agree or 7 strongly agree)
- AND there was no more than 1 outlier in the opposite direction (answer 3/2/1)

Answer “0” by any of the panelists was not included as an obstacle to consensus achievement, given the multidisciplinary nature of the consensus and the voting panel.

Surveys were distributed to authors using Google Forms (Google, Mountain View, CA). For every statement, a free text option was available to state the reason the panelist disagreed and an amendment suggestion; all answers were anonymized.

## 3 Results

Characteristics of the included faculty for this consensus are summarized in Table 1.

**TABLE 1:** Characteristics of the included faculty for this consensus.

	N over 44 experts
Specialization	
Otolaryngologists and head and neck surgery	19 (43.2%)
Oncology	14 (31.8%)
Radiation oncology	11 (25%)
Continent of activity	
Europe	31 (70.5%)
North America	8 (18.2%)
South America	3 (6.8%)
Asia	2 (4.5%)
Age (years)	
30–40	7 (15.9%)
41–50	17 (38.6%)
51–60	12 (27.3%)
> 60	8 (18.2%)
Professional experience in sinonasal and skull base tumors (years)	
< 5	2 (4.5%)
6–10	7 (15.9%)
11–20	20 (45.5%)
21–30	10 (22.7%)
> 31	5 (11.4%)

**FIGURE 1:**
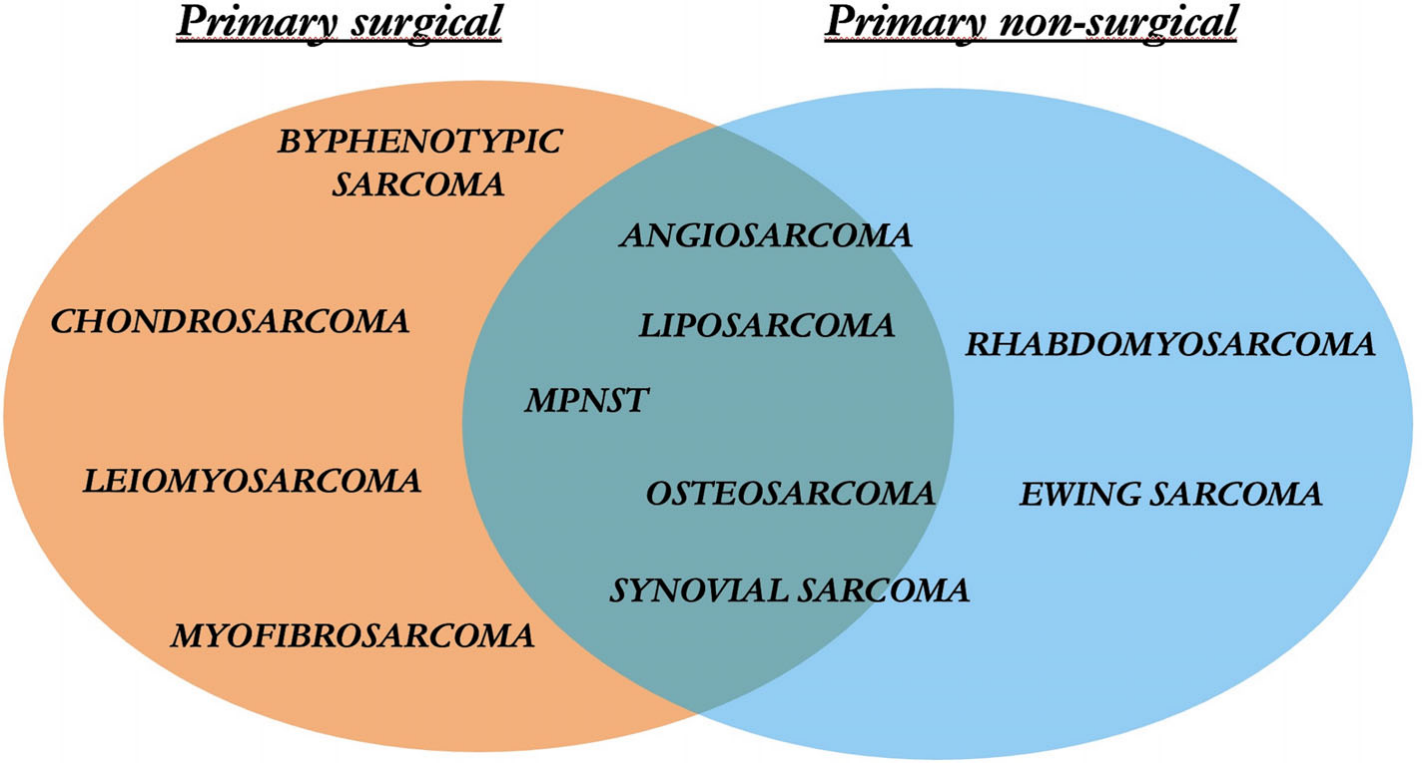
Overview of primary treatment strategies by histological subtype in sinonasal sarcomas. The diagram illustrates the initial management approach, either primary surgical or non‐surgical, associated with various sarcoma subtypes. MPNST = Malignant peripheral nerve sheath tumor.

Initially, the 62 statements were sent, and consensus was reached in 33/62 (53.2%) statements. The remaining statements were reviewed following comments given by the panelists. Of the remaining 29/62 items, 3 were unified, and 2 added, leading to 28 “second‐round” statements. Consensus was subsequently reached in all cases (100%) (Tables 2 and 3; Figure 1).

**TABLE 2:** Statements that reached consensus for sinonasal sarcomas management.

Statement code	Statement	Rate of consensus (round)	Outliers (1/2/3)	Unknown answers (0)
**SESSION 1 ‐ SINONASAL SARCOMAS: GENERAL CONSIDERATION**				
S1.1	Sinonasal sarcomas are rare entities whose histopathological diagnosis should be centralized in tertiary referral centers and based on immunohistochemical and genetic analyses.	93.6% (first)	1	0
S1.2	Whenever feasible, sinonasal sarcomas treatment is based on multimodal therapies with surgery being, in the majority of cases, the cornerstone of treatment.	91.3% (first)	0	1
S1.3	Contrary to guidelines on extremity sarcomas, wide surgical margins (> 4 cm) are generally difficult to achieve during the surgical management of these endonasal tumors, leaving the appropriateness of surgical margins open to debate.	91.3% (first)	1	1
S1.4R	Taking into consideration that wide surgical margins (> 4 cm) are generally difficult to achieve during the surgical approach, adjuvant radiation therapy (IMRT or PBRT) seems necessary for local control also in case of negative resection margins.	87.8% (second)	1	0
S1.5R	Even if a multidisciplinary discussion is always required, adjuvant radiation therapy can be avoided in selected cases of low‐grade sarcomas not extending beyond the sinonasal compartment, surgically resected with negative microscopic margins (R0).	88.1% (second)	1	0
S1.6R	In case of sarcomas not presenting regional or distant metastasis, the tumor grade, stage, histology and surgical free resection margins represent the main prognosticators for sinonasal sarcomas.	100% (second)	0	0
**SESSION 2 ‐ ANGIOSARCOMA**				
S2.1R	Given the challenges of sinonasal angiosarcoma resection and its systemic and often multifocal nature, systemic chemotherapy can be used in a neoadjuvant setting. However, in resectable cases, surgery with free margins (upfront or after induction chemotherapy) should be considered the curative treatment of choice.	92.5% (second)	1	2
S2.2R	Adjuvant radiotherapy (IMRT) is frequently indicated if no contraindications are present and/or in case of a low‐grade tumor with free resection margin surgery.	86.8% (second)	1	4
S2.3R	In unresectable cases, a combination of radiotherapy (IMRT/IMPT) and chemotherapy is usually the treatment of choice.	100 (second)	0	0
S2.4R	In metastatic disease, chemotherapy is the treatment of choice and can be associated, based on a multidisciplinary discussion, with ‐ Surgery: in case of a single and easily resectable metastasis ‐ RT (IMRT): in case of an unresectable symptomatic single metastasis or oligometastatic disease (SBRT with a palliative intent)	97.5% (second)	0	1
S2.5R	Chemotherapy in a neoadjuvant/adjuvant setting is generally based on taxanes and/or anthracyclines, with and emerging role for antiangiogenics, notably tyrosine kinase inhibitors (TKIs), and immune checkpoint inhibitors (ICIs).	94.1% (second)	0	6
**SESSION 3 ‐ BIPHENOTYPIC SINONASAL SARCOMA**				
S3.1	In resectable cases, surgery with free margins represents the front‐line treatment of choice	100% (first)	1	1
S3.2R	Adjuvant radiotherapy (IMRT or PBRT) is reserved for cases with R1‐R2 margins, perineural or lymphovascular invasion and should be discussed in R0 high‐stage disease.	92.8% (second)	1	0
S3.3	In case of local (more frequent) or distant (rare) recurrence, surgery still represents the treatment of choice	80.4% (first)	1	1
S3.4	Induction or adjuvant chemotherapy is not indicated a priori, considering its low‐grade nature	91.3% (first)	1	1
S3.5	In unresectable cases, radiotherapy (IMRT or PBRT) represents the treatment of choice	91.3 (first)	1	1
**SESSION 4 ‐ CHONDROSARCOMA**				
S4.1	Surgery with free margins represents the front‐line treatment of choice	91.3% (first)	0	1
S4.2R	In the case of locally advanced tumor with vital structures involvement, the R1/R2 surgery should be discussed taking into consideration expected post‐operative morbidities.	88.1% (second)	1	0
S4.3R	After the primary surgical approach, adjuvant radiotherapy (IMRT or PBRT) should be multidisciplinary discussed and proposed to R1‐R2 margins unresectable tumors or selected R0 margins tumors, with high‐risk features.	85.7% (second)	1	0
S4.4R	IMPT provides better local control outcomes compared to IMRT, even if it lacks conclusive evidence.	87.5% (second)	0	1
S4.5	Mesenchymal and dedifferentiated chondrosarcoma is thought to have increased chemosensitivity with a higher propensity to recur and metastasize. As part of multimodal therapy, chemotherapy is usually applied in neoadjuvant or adjuvant scenarios.	92.5% (first)	0	7
**SESSION 5 ‐ EWING SARCOMA**				
S5.1	Neoadjuvant chemotherapy with 3‐drugs or 4‐drugs regimen is often indicated as front‐line treatment in order to down‐stage the primary tumor.	91.3% (first)	1	1
S5.2	Patients with stable or decrease tumor volume after primary neoadjuvant treatment should receive local control therapy based on ‐ Surgical excision: if possible, based on tumor extension and patient comorbidities. ‐ Radiotherapy (IMRT or PBRT): if surgery is contraindicated.	95.6% (first)	1	1
S5.3	Adjuvant chemotherapy after surgical resection is generally recommended regardless of surgical margins.	100% (first)	0	1
S5.4	Adjuvant postoperative radiotherapy (IMRT or PBRT) is recommended in case of R1‐R2 surgical margins.	88.8% (first)	1	2
S5.5	Patients with progressive disease after primary neoadjuvant chemotherapy should receive second lines chemotherapy associated with radiotherapy/surgery of the primary site for local control or palliation, based on a multidisciplinary discussion.	84.7% (first)	1	1
S5.6	Relapsed or refractory disease should be treated with chemotherapy with or without radiotherapy (IMRT or PBRT), based on tumor extension.	93.3% (first)	0	2
**SESSION 6 ‐ LEIOMYOSARCOMA**				
S6.1	In resectable cases, surgery with free margins represents the front‐line treatment of choice.	88.6% (first)	1	3
S6.2R	Adjuvant radiotherapy (IMRT) after the primary surgical approach should be multidisciplinary discussed and proposed to R1‐R2 margins unresectable tumors or selected high‐grade R0 margins tumors.	95.1% (second)	0	1
S6.3	In unresectable cases, the application of neo‐adjuvant chemotherapy followed by surgery or palliation radiotherapy (IMRT) should be discussed case‐by‐case.	93.0% (first)	0	4
S6.4R	In metastatic cases, chemotherapy represents the treatment of choice; local therapies should be multidisciplinary discussed.	100% (second)	0	0
**SESSION 7 ‐ LIPOSARCOMA**				
S7.1	In resectable cases, surgery with free margins represents the front‐line treatment of choice.	95.5% (first)	0	2
S7.2	Adjuvant radiotherapy (IMRT or PBRT) is indicated in case of R1‐R2 margins and/or high‐grade tumor and/or large local extension.	91.1% (first)	0	2
S7.3	Neoadjuvant/adjuvant chemotherapy is not indicated a priori but can be discussed in case of high‐grade liposarcomas and/or unresectable/metastatic cases.	88.8% (first)	0	2
**SESSION 8 – LOW‐GRADE MYOFIBROBLASTIC SARCOMA (MYOFIBROSARCOMA)**				
S8.1	In resectable cases, surgery with free margins represents the front‐line treatment of choice.	97.7% (first)	0	3
S8.2	In unresectable cases, the application of neo‐adjuvant chemotherapy followed by surgery or palliation radiotherapy (IMRT) should be discussed case‐by‐case.	88.3% (first)	0	4
S8.3	In metastatic cases (regional or distant), surgery associated with radiotherapy (IMRT) still represents the treatment of choice, with chemotherapy reserved to selected cases due to its general low response rate.	83.7% (first)	1	4
**SESSION 9 ‐ MALIGNANT PERIPHERAL NERVE SHEAT TUMOR**				
S9.1	In resectable cases, surgery with free margins represents the front‐line treatment of choice.	88.6% (first)	1	3
S9.2R	Adjuvant radiotherapy (IMRT) is generally indicated in R1‐R2 margins, high‐grade tumor and/or large local extensions. Its application in R0 cases should be discussed case‐by‐case.	87.5% (second)	0	2
S9.3	Adjuvant chemotherapy after surgical excision is controversial and mainly indicated in high‐grade tumor.	90.9% (first)	0	3
S9.4R	In unresectable cases, palliative radiotherapy (IMRT/IMPT) vs. palliative surgery followed by radiation therapy should be discussed case‐by‐case.	100% (second)	0	2
S9.5	In metastatic cases (rare), chemotherapy represents the treatment of choice, primarily based on doxorubicin + ifosfamide regimen, even if with scarce results.	85.3% (first)	1	6
**SESSION 10 ‐ OSTEOSARCOMA**				
S10.1R	In resectable cases, surgery with free margins represents the front‐line treatment of choice, with neo‐adjuvant chemotherapy reserved for high‐grade tumors.	95.2% (second)	1	0
S10.2R	Adjuvant radiotherapy (IMRT or PBRT) is primarily indicated in R1‐R2 margins and should be discussed in R0 margins cases, based on tumors grade.	95.2% (second)	0	0
S10.3R	Adjuvant chemotherapy is a possible solution, in addition to radiotherapy, in high‐grade tumors or in positive resection margins (R1‐R2) tumors.	90.2% (second)	0	1
S10.4R	In unresectable tumors, a combination of chemotherapy and radiotherapy (IMRT or IMPT) represents the treatment of choice.	95.2% (second)	0	0
S10.5	Chemotherapy is the treatment of choice of metastatic cases.	95.5% (first)	O	2
**SESSION 11 – RHABDOMYOSARCOMA**				
S11.1A	Induction chemotherapy is considered the upfront treatment of choice in most cases.	100% (second)	0	0
S11.2A	A chemo‐radiotherapy (IMRT/IMPT) approach is the treatment of choice after induction chemotherapy.	92.8% (second)	1	0
S11.3A	In responders to chemotherapy with a localized lesion, surgical resection with free margin should be considered and evaluated case‐by‐case.	90.4% (second)	1	0
S11.4	The most used chemotherapy regimens are vincristine, actinomycin D, and cyclophosphamide (VAC); vincristine, dactinomycin, and ifosfamide (VAI); vincristine, ifosfamide, and etoposide (VIE).	88.6% (first)	1	3
S11.5	Intrathecal chemotherapy is no longer indicated in patients with evidence of tumoral cells inside the CSF.	82.8% (first)	1	12
S11.6R	Most patients with nonmetastatic resectable relapse can receive a locoregional treatment of surgery or RT (IMRT/IMPT), based on previous protocols, associated with adjuvant/neoadjuvant chemotherapy.	100% (second)	0	0
S11.7	In case of unresectable recurrence, re‐irradiation (IMRT) with second‐line chemotherapy should be discussed.	86.9% (first)	1	1
S11.8	In case of metastatic pathology, chemotherapy is indicated based on previous treatments received.	97.7% (first)	0	2
S11.9	In pleomorphic rhabdomyosarcoma, due to its nature being more common to other soft tissue sarcomas, it is generally treated with upfront surgery +/− radiotherapy (IMRT) based on the margin status and histologic analysis	83.7% (first)	1	4
**SESSION 12 – SYNOVIAL SARCOMA**				
S12.1R	Surgery is the mainstay of treatment of sinonasal synovial sarcomas; however, chemotherapy can be used in neo‐adjuvant setting and should be discussed case‐by‐case.	95% (second)	0	2
S12.2R	Adjuvant radiotherapy (IMRT or PBRT) can improve local control both in R1‐R2 margins and R0 cases with high‐risk features, based on a multidisciplinary discussion.	97.5% (second)	0	1
S12.3R	Unresectable cases are generally treated with a combination of chemotherapy and radiotherapy (IMRT or PBRT).	97.6% (second)	0	0
S12.4R	In metastatic cases, chemotherapy (anthracycline‐based regimens) is the treatment of choice.	97.4% (second)	0	3
S12.5	Pazopanib could be administrated in subsequent lines.	89.4% (first)	1	9

*Note: Statement code legend: “A” indicates that the statement has been added by the Scientific Committee based on the feedback received during the first round; “R” indicates that the statement has been revised*.

Abbreviations: IMPT = Intensity Modulated Proton Therapy, IMRT = Intensity Modulated Radiation Therapy, PBRT = Particle‐Beam Radiation Therapy, RT = radiotherapy, SBRT = Stereotactic Body Radiation Therapy.

**TABLE 3:** Overview of treatment strategies and corresponding levels of evidence for SNS histologic subtypes.

Histology	Upfront treatment strategy	Level of evidence
Angiosarcoma	Surgery or neoadjuvant CHT in selected cases	4
Biphenotypic Sarcoma	Surgery	4
Chondrosarcoma	Surgery	4
Ewing Sarcoma	Induction CHT followed by surgical resection and/or RT	4
Leiomyosarcoma	Surgery	4
Liposarcoma	Surgery or neoadjuvant CHT for high grade tumour	4
Low‐Grade Myofibroblastic Sarcoma (Myofibrosarcoma)	Surgery	4
Malignant Peripheral Nerve Sheat Tumor	Surgery	4
Osteosarcoma	Surgery or neoadjuvant CHT for high grade tumour	4
Rhabdomyosarcoma	Induction CHT followed by CHT + RT or surgery	Role of neoadjuvant CHT: 2 Role of RT: 3 Role of surgery: 4
Synovial Sarcoma	Surgery or neoadjuvant CHT in selected cases	4

*Note*: Levels of evidence are reported according to the Oxford Centre for Evidence‐Based Medicine (OCEBM, 2011): Level 1 = systematic reviews of randomized trials, Level 2 = individual randomized trials or observational studies with dramatic effect, Level 3 = non‐randomized controlled cohort/follow‐up studies, Level 4 = case‐series or low‐quality cohort/case‐control studies, Level 5 = expert opinion without explicit critical appraisal or based on basic science.

Abbreviations: CHT = chemotherapy, RT = radiotherapy.

## 4 Discussion

SNS are a heterogeneous group of malignant tumors for which standardized treatment protocols are lacking due to the different histologies and rarity of each subtype [9], leaving the management of these entities to the center to which the patient refers (Supporting Information Session 1.1). Recently, the International Consensus Statement on Allergy and Rhinology has provided a broad overview of sinonasal malignancies, including the main sarcoma subtypes. Nevertheless, the present work is specifically dedicated to SNS, offering a multicentric international consensus that provides clear indications, in the absence of studies of greater significance, in the management of each SNS, making this work the first of its kind [10].

The general concept of SNS management approved by the present consensus is that multimodal strategies are needed, and surgery (principally endoscopic endonasal) with negative margins represents the cornerstone of treatment for most sarcomas (Supporting Information Sessions 1.2 and 1.3) [11, 12]. Nevertheless, on one hand, the upfront surgical management is reserved for specific histologies; on the other hand, it must be considered in the multimodal approach to SNS. However, experts concurred that surgery with clear margins > 4 cm would lead to an unacceptable level of morbidity, so that adjuvant radiotherapy (RT) with photons (IMRT) or ion beams (IMPT) seems generally necessary for local control, even if a multidisciplinary discussion is always required (Supporting Information Sessions 1.4R and 1.5R). The SNS final prognosis is mainly influenced by tumor grade/stage, histology, and surgical free resection margins (Supporting Information Session 1.6R) [13, 14].

In accordance with the histologically driven approach, typical of epithelial tumors [15] and the result of this international consensus, each SNS subtype will be examined separately and discussed based on its more frequent upfront management (surgical, non‐surgical, or multimodal).

### 4.1 Upfront Surgical Treatment

#### 4.1.1 Biphenotypic Sarcoma

Biphenotypic sarcoma is a low‐grade sinonasal tumor with a slow and infiltrative pattern of growth with no tendency to distant metastasis, but high propensity for local recurrence [16].

Absolute consensus was achieved that the upfront treatment of choice for this tumor is free‐margin surgical excision (Supporting Information Session 3.1), [17] and adjuvant RT (IMRT or IMPT) is reserved in case of close or positive margins, perineural spread, lymphovascular invasion, and involvement of difficult to resect areas (cavernous sinus, orbital apex) (Supporting Information Sessions 3.2R and 3.5). In the case of local recurrence, surgery still represents the treatment of choice (Supporting Information Session 3.3) [18]. The role of CHT is controversial, and the rare metastasis reported in the literature have been treated with surgical resection (Supporting Information Session 3.4).

The 5‐year overall survival (OS) and disease‐free survival (DFS) rates are 100% and 80 ± 17.9%, respectively [18].

#### 4.1.2 Chondrosarcoma

Chondrosarcomas include several subtypes, with the conventional form being the most common (80%), and the clear cell, mesenchymal, and dedifferentiated variants constituting the remaining 20% [19, 20].

Experts concurred that surgery with free margins represents the treatment of choice both for primary and recurrent cases of sinonasal chondrosarcomas; however, truly radical surgery can be virtually impossible to obtain in extensive local disease, a frequent scenario due to the common indolent tumor growth and proximity to vital structures (Supporting Information Session 4.1) [21, 22, 23]. In particular, sinonasal chondrosarcomas are often more amenable to complete surgical resection with negative margins, whereas skull base tumors frequently extend to critical areas such as the orbital apex, cavernous sinus, orbit, or internal carotid artery, making radical surgery and margin‐free resections far more challenging. In this context, a “maximum safe resection” is the mainstay of upfront treatment, followed by RT (Supporting Information Session 4.2R) [24, 25].

The latter adjuvant treatment should be multidisciplinary discussed and proposed to R1–R2 margins unresectable tumors or selected R0 margins tumors with high‐risk features, due to its positive effect on local control, specifically in evolutive disease (Supporting Information Session 4.3R) [26, 27]. Nevertheless, sinonasal chondrosarcomas have been historically considered relatively radioresistant, and their post‐operative application has the goal to slow down the growth of any residual tumor [19]. Overall, from the present consensus, IMPT seems to be more effective than IMRT with an OS benefit (Supporting Information Session 4.4R) [28, 29].

CHT does not have a role in chondrosarcomas management, except for cases of rare mesenchymal or dedifferentiated forms, which present an increased propensity to recur and metastasize, for which CHT can be applied in a neoadjuvant or adjuvant setting based on the multidisciplinary discussion (Supporting Information Session 4.5) [26, 30].

The overall 5‐ and 10‐year OS are, respectively, 83.3%–93.1% and 44%–70% [23, 24].

#### 4.1.3 Leiomyosarcoma

Leiomyosarcoma is an aggressive neoplasia derived from the smooth muscle cells located in the tunica media of blood vessels [31, 32]. Similar to the above‐mentioned histologies, experts concurred that the primary treatment of choice for leiomyosarcoma is complete surgical resection, with RT (IMRT) reserved for patients with locally advanced, recurrent, or high‐grade tumors (Supporting Information Sessions 6.1 and 6.2R) [31, 33]. Even if data on CHT in the adjuvant setting are inconclusive, this consensus suggests that it can be used in case of unresectable or metastatic disease to shrink the tumor, and it can be associated with adjuvant therapies (surgery or RT) (Supporting Information Sessions 6.3 and 6.4R) [32, 33].

The OS reported in a recent systematic review was 66% at 38.2 months; therefore, the overall 5‐year survival rate (in 10 patients) was 20% [32].

#### 4.1.4 Low‐Grade Myofibroblastic Sarcoma (Myofibrosarcoma)

Myofibrosarcoma is a low mitotic rate tumor that has replaced, in the WHO fifth classification, the sinonasal fibrosarcoma; however, the management of this tumor derives from the experience of sinonasal fibrosarcoma due to the overlapping clinical behavior [34].

Strong consensus was achieved regarding the upfront treatment of this tumor, which is usually surgical, with radiotherapy applied in an adjuvant setting only in selected cases (i.e., positive margins) (Supporting Information Sessions 8.1 and 8.2) [35, 36]. CHT is generally not required since myofibrosarcoma cells have shown established co‐resistance to several chemotherapeutic agents and present a low response rate [34, 35]. However, the application of anthracyclines in neo‐adjuvant or adjuvant settings is always a possibility and should be discussed in unresectable or metastatic cases, even if, for the latter, surgery associated with radiotherapy should be considered the treatment of choice (Supporting Information Session 8.3) [37, 38].

The 5‐year OS is 56.6%–77.8% [38].

### 4.2 Upfront Non‐Surgical Treatment

#### 4.2.1 Ewing Sarcoma

Ewing sarcoma is a pathologic entity in the context of small‐blue‐round‐cell malignant tumors, with the adamantinomatous variant typical of the sinonasal tract [39, 40, 41].

Systemic CHT is an important component of the Ewing sarcoma management since it is used both in the neoadjuvant and adjuvant settings [39]. Specifically, as described by recent papers and strongly confirmed by this consensus, CHT is used both before and after local therapies to downsize the tumor, increasing the probability of free resection margins, and decreasing the metastatic risk (Supporting Information Sessions 5.1 and 5.3) [42, 43].

Patients with improved or stable disease after induction CHT should receive local control with curative intent. Up to date, no randomized studies have compared surgery versus RT; however, several clinical trials seemed to prefer radical surgery due to the better local control rates and OS impact [39]. However, some have suggested that in anatomic locations, not amenable to surgery with wide margins (such as the sinonasal compartment), definitive RT may be an effective treatment [40, 44]. Given this evidence, experts of this consensus concurred that the surgical excision of sinonasal Ewing sarcoma should be preferred to RT, whenever feasible, with the latter (IMRT or PBRT) reserved when surgery is contraindicated or in addition to surgery in case of positive resection margins (Supporting Information Sessions 5.2 and 5.4).

Poor response to upfront neoadjuvant CHT has been identified as an adverse prognostic factor in non‐metastatic disease, so that the local management of these scenarios should be carefully evaluated [45, 46]. The benefit of an aggressive surgical approach that does not result in OS improvement should be discussed with other alternatives, such as local control with RT.

In the setting of recurrence, usually metastatic, second‐line options must be considered (Supporting Information Sessions 5.5 and 5.6) [39].

The review by Shaari et al. on sinonasal Ewing sarcoma found an OS of 60% at 42 months [47].

#### 4.2.2 Rhabdomyosarcoma

Sinonasal rhabdomyosarcoma presents different subtypes: Embryonal (80%), alveolar (15%–20%), pleomorphic (2%–4%), and spindle‐cell type, extremely rare in the sinonasal region [17]. Pediatric cases are predominantly embryonal with higher chemosensitivity, whereas adult cases more often show alveolar or pleomorphic histology, generally associated with variable prognosis. The multimodal treatment strategies for this tumor are based on a specific risk stratification system that not only considers the TNM system, but also histology, fusion status (PAX3 or 7/FOXO1), anatomical site (favorable vs. unfavorable), size, and age [48, 49].

As proposed by the Intergroup Rhabdomyosarcoma Study Group trials, experts of this consensus agreed that the primary treatment modality of this tumor is represented by multi‐agent CHT (VAC, VAI, or VIE, Supporting Information Session 11.4) since this tumor presents high chemosensitivity, particularly in the embryonal and alveolar subtypes (Supporting Information Sessions 11.1A and 11.5) [50]. Upfront CHT often determines tumor shrinkage, which facilitates local therapies and ultimately improves survival rates [51]. Given the critical surrounding structures, tumor characteristics, and radiosensitivity, CHT‐RT is often preferred as local treatment over attempts at surgical resection [49]. The prescription dose and modality (IMRT vs. IMPT) depend on the histotype, site of the primary tumor, and nodal involvement (Supporting Information Session 11.2A); nevertheless, data from a pooled analysis of over 1100 patients with parameningeal disease showed that those who received RT had a significantly higher OS rate (68.5%) compared to those who did not (40.8%). This suggests that avoiding RT may come at the cost of reduced survival and local control [52].

Surgery has recently been described as beneficial in the multimodal management of rhabdomyosarcomas, not only to debulk lesions with compressive symptoms, but also as an adjuvant approach, particularly in cases in which free resection margins are achievable (Supporting Information Session 11.3A) [53]. The survival benefit of surgery in the multimodal treatment of rhabdomyosarcoma has been clearly shown in numerous independent studies; however, the extension of surgery should be tailored to each patient and be as functional as possible [48, 54]. Some authors also suggest that surgery can be used as upfront treatment, in really selected cases of early‐stage surgically resectable tumors or in cases of pleomorphic variant (Supporting Information Session 11.9) [53, 55]. In addition to this, salvage surgery is critical for survival in patients who relapse locally after definitive chemoradiation (Supporting Information Session 11.6R) [53, 56].

In the case of unresectable recurrence or metastatic disease, consensus was achieved on the importance of chemotherapeutic regimens that represent the treatment of choice [57]; nevertheless, in non‐metastatic disease, it is important to achieve a complete local disease control through surgery and/or re‐irradiation, since no locoregional treatment often results in death from progression of disease (Supporting Information Sessions 11.7 and 11.8) [57].

The 5‐year OS is 40%–45%, female patients and those with alveolar type rhabdomyosarcoma presented a better prognosis [17].

### 4.3 Upfront Multimodal Approach

#### 4.3.1 Angiosarcoma

Sinonasal angiosarcoma is a highly aggressive malignant tumor originating from lymphatic or vascular endothelial cells.

Upfront surgical resection is the cornerstone of treatment of this tumor; however, due to its rapid progression and metastatic nature, surgery is not always applied as primary treatment [58]. Indeed, experts of this consensus agreed that, even if few case series are available, neoadjuvant CHT can be used in selected cases, reserving surgery only in case of tumors with potential negative resection margins (Supporting Information Session 2.1R) [58]. Because of the high risk of local recurrence, adjuvant RT is often recommended, underlying the importance of local disease control to ameliorate OS (Supporting Information Session 2.2R) [59]. Nevertheless, despite a substantial risk of subsequent metastatic disease, there are no evidence that adjuvant CHT is beneficial; indeed, as underlined by this consensus, CHT is usually applied only in unresectable cases and metastatic disease, with anthracyclines, ifosfamide, and taxanes being the most used drugs, and biological therapies (i.e., antiangiogenics and immunotherapy) that offer hope for future therapies (Supporting Information Session 2.5R) [60]. Considering that angiosarcoma has been found to have an overall response rate of 26%–63% to CHT [59, 60], experts confirmed that even in metastatic disease, adjuvant local therapies (surgery or RT) are often indicated in oligometastatic disease (Supporting Information Sessions 2.3R and 2.4R).

The 5‐year OS is 35%, even with localized disease [59].

#### 4.3.2 Liposarcoma

Liposarcoma is an aggressive lipomatous tumor that presents different histological subtypes: Well‐differentiated, dedifferentiated, myxoid, pleomorphic, and not otherwise specified [17].

According to the available literature, this consensus agrees that upfront surgery is the treatment of choice, with RT (IMRT or PBRT) reserved for positive margins or high‐grade tumors (Supporting Information Sessions 7.1 and 7.2) [61, 62]. Particle therapy may also be discussed in the treatment of lesions difficult to resect and in those who have refused surgery for potential post‐surgical functional issues [63].

CHT (adjuvant or neoadjuvant) is not indicated a priori but must be discussed multidisciplinarily in the case of high‐grade liposarcomas or unresectable/metastatic cases (Supporting Information Session 7.3) [63].

The 5‐year OS of head and neck liposarcoma was 66%–83% [62].

#### 4.3.3 Malignant Peripheral Nerve Sheath Tumor (MPNST)

MPNST is one of the most aggressive malignant lesions in the head and neck area, with a particularly high rate of local recurrence [64].

Based on the available data, experts consider complete surgical removal the mainstay of treatment and key element for survival; patients in whom a total tumor resection was not feasible showed worse outcomes, with a mean survival of 19 months, compared with 30 months for patients whose tumors could be resected completely (Supporting Information Session 9.1) [64].

Historically, MPNST was considered radioresistant, probably due to older radiotherapeutic techniques rather than biologic behavior. Indeed, recent data suggest that adjuvant RT should be considered in selected cases with encouraging results (Supporting Information Session 9.2R) [65].

Conversely, the role of systemic CHT remains controversial (Supporting Information Sessions 9.3, 9.4R, and 9.5) [66].

The 5‐year OS in the head‐and‐neck region was 15% [67].

#### 4.3.4 Osteosarcoma

Gnathic or craniofacial osteosarcomas management generally requires surgery with free margins; however, when it occurs in the sinonasal cavity, on one hand, the anatomical constraints make this surgical practice often difficult to obtain, on the other hand, the significant proportion of young people affected by osteosarcomas makes disfiguring surgery contraindicated [68]. Nevertheless, primary bone malignancies are known to be relatively radioresistant tumors, and historically, there has been a limited role for RT [69]. Considering that local recurrence is by far the most common pattern of failure in sinonasal osteosarcoma (94%), in a recent review, the role of RT was found to be applied in an adjuvant setting in the majority of cases, with non‐conclusive results on local control and OS [68]. In addition, the role of CHT has been investigated and found, by some authors [70, 71], to be particularly efficient in intermediate/high‐grade osteosarcomas since it provides better local control, although this effect was not shown in OS and DFS [72].

As a result, this consensus confirmed the primary role of upfront surgery (Supporting Information Session 10.1R) followed by radiotherapy in selected cases (Supporting Information Session 10.2R), given the uncertain role of this adjuvant therapy. CHT should be reserved in the neo‐adjuvant setting in high‐grade tumors (Supporting Information Session 10.1R), or in the adjuvant setting in case of metastasis (rare a priori) or unresectable tumors (Supporting Information Sessions 10.3R, 10.4R, and 10.5R).

The 5‐year OS rate was 35%–55% [68].

#### 4.3.5 Synovial Sarcoma

Synovial sarcoma takes its name from the propensity to develop in the deep soft tissue of extremities; however, these tumors do not derive from synovial cells and seem to originate from primitive mesenchymal stem cells [73].

Its main treatment is represented by complete surgical resection [74]. Nevertheless, interestingly, it has a relatively good response to CHT, so that experts concurred that neoadjuvant CHT should be discussed case‐by‐case (Supporting Information Session 12.1R). This finding is based on several studies in the 1990s that reported good response to anthracycline‐based regimens not only in unresectable or metastatic cases (Supporting Information Session 12.3R and 12.4R) but also in the neoadjuvant setting [75].

RT (IMRT or PBRT) seems to improve local control and OS of this sarcoma, specifically in high‐grade lesions, leaving its indication to multidisciplinary discussion (Supporting Information Session 12.2R) [76].

In the management of synovial sarcoma after first‐line CHT failure, Pazopanib can be used as a treatment option in advanced or metastatic cases, due to its multi‐targeted tyrosine kinase inhibitor directed against the receptor tyrosine kinases (RTKs), vascular endothelial growth factor (VEGFR) 1/2/3, platelet‐derived growth factor (PDGFR), thereby inhibiting tumor growth and angiogenesis (Supporting Information Session 12.5) [77].

Five‐year local control is reported at 86%, while 5‐years OS is 82% [76].

#### 4.3.6 Study Limitations

This consensus is limited by the rarity and heterogeneity of SNS; many of these tumors are exceedingly rare, and the statements rely on very limited literature in these areas (many of which referred to head and neck collectively), which further constrains the strength of the recommendations. In addition, the inclusion of newly recognized entities, such as low‐grade myofibroblastic sarcoma, reflects recent advances but may challenge reproducibility across institutions with limited expertise.

Undifferentiated sarcoma was not included in the present consensus due to its WHO fifth ed. histological definition, “heterogeneous group of neoplasms without any identifiable line of differentiation,” which renders this histology of unknown origin and with a frequent diagnosis of exclusion. Moreover, the authorship is predominantly from Europe (70%) and the United States, with limited input from South America, Asia, and Africa, which may affect global generalizability. Finally, while the Delphi approach enables expert consensus, it often lacks evidence‐based standards of care, which should be acknowledged as an additional limitation.

## 5 Conclusions

The management of SNS remains one of the most complex challenges in head and neck oncology, due to their rarity, anatomical location, and biological heterogeneity. This international consensus emphasizes the central role of a histology‐driven approach in guiding upfront treatment decisions, aligning therapeutic strategies with the unique biological behavior of each sarcoma subtype; nevertheless, even within this structured framework, SNS exhibit highly variable prognosis.

The creation of an international, multi‐institutional registry dedicated to SNS could represent a critical step forward to collect large‐scale data and strengthen the evidence base for future treatment strategies.

## Author Contributions

AV, MTZ and PB contributed to the conceptualization, methodology, project administration, and supervision of the study. AV, MTZ and PB were responsible for data curation. AV, FC, MV, PB and PH contributed to data visualization. Additional contributions to conceptualization were provided by SP, JYB, AI, EO, LL, AMC, PS, DMT, BK, JMB, JT, and PGC. Methodology contributions also included MF, EWW, CM, SH, AG, FABC, NH, CV, AD, CG, PH, and EYH. Data curation was also supported by AMC, DG, AS, PN, ID, MC, SH, AG, SYS, and ML.

All authors contributed equally to validation, investigation, resources, writing, review, and editing of the manuscript. Supervision involved PH, EYH, and LL.

## Disclosure

The authors have nothing to report.

## Conflicts of Interest

C.S. reports advisor role for Astrazeneca, DMT reports consulting for Boston Scientific and Servier (unrelated).

## Supporting information




**Supporting file 1**: alr70038‐sup‐0001‐SuppMat.docx
