# Supplementary material for: Sinonasal Sarcomas Management: An International Consensus Statement
Source: Int Forum Allergy Rhinol. 2025 Sep 30;16(1):55–69. doi: 10.1002/alr.70038 (PMC12761347; doi:10.1002/alr.70038)
Supplement: Supplementary file 1 — Supporting file 1: alr70038‐sup‐0001‐SuppMat.docx [file ALR-16-55-s001.docx]

Consensus Conference on Sinonasal Sarcomas Management

Statements list

**INDEX**

[SESSION 1 - Sinonasal sarcomas: general consideration 2](#_Toc202198207)

[SESSION 2 - Angiosarcoma 3](#_Toc202198208)

[SESSION 3 - Biphenotypic sinonasal sarcoma 5](#_Toc202198209)

[SESSION 4 – Chondrosarcoma 6](#_Toc202198210)

[SESSION 5 – Ewing sarcoma 7](#_Toc202198211)

[SESSION 6 – Leiomyosarcoma 8](#_Toc202198212)

[SESSION 7 – Liposarcoma 9](#_Toc202198213)

[SESSION 8 – Low-grade myofibroblastic sarcoma (Myofibrosarcoma) 9](#_Toc202198214)

[SESSION 9 – Malignant peripheral nerve sheat tumor 10](#_Toc202198215)

[SESSION 10 – Osteosarcoma 11](#_Toc202198216)

[SESSION 11 – Rhabdomyosarcoma 13](#_Toc202198217)

[SESSION 12 – Synovial sarcoma 15](#_Toc202198218)

List of acronyms:

- CSF: cerebrospinal fluid
- IMPT: intensity modulated proton therapy
- IMRT: intensity modulated radiotherapy
- PBRT: proton beam radiotherapy
- RT: radiotherapy
- SBRT: Stereotactic body radiation therapy

**Response rate:** refers to the number of valid responses (ratings from 1 to 7) provided for each statement. Responses marked as unknown answer (0) are excluded from this count.

**Rate of consensus:** consensus is considered reached only when at least 80% of participants select one of the agreement options (6=*agree*; 7=*strongly agree)*

**Outliers**

Refers to the number of responses in the opposite direction of agreement (1=*strongly disagree*; 2=*disagree*; 3= *somewhat disagree*)

# **SESSION 1 - Sinonasal sarcomas: general consideration**

**S1.1 - Sinonasal sarcomas are rare entities whose histopathological diagnosis should be centralized in tertiary referral centers and based on immunohistochemical and genetic analyses.** (1)

Response rate: 47/47

Rate of consensus: 93.6%

Outliers: 1

Statement approved? Yes

**S1.2 - Whenever feasible, sinonasal sarcomas treatment is based on multimodal therapies with surgery being, in the majority of cases, the cornerstone of treatment.** (2,3)

Response rate: 46/47

Rate of consensus: 91.3%

Outliers: 0

Statement approved? Yes

**S1.3 - Contrary to guidelines on extremity sarcomas, wide surgical margins (> 4 cm) are generally difficult to achieve during the surgical management of these endonasal tumors, leaving the appropriateness of surgical margins open to debate.** (2,3)

Response rate: 46/47

Rate of consensus: 91.3%

Outliers: 0

Statement approved? Yes

**S1.4 - Free resection margins did not represent a predictive factor for achieving a complete therapeutic response, and radiation therapy (IMRT or PBRT) seems necessary, a priori, for local control.** (4–6)

Response rate: 45/47

Rate of consensus: 71.7%

Outliers: 5

Statement approved? No

**S1.4R - Taking into consideration that wide surgical margins (> 4 cm) are generally difficult to achieve during the surgical approach, adjuvant radiation therapy (IMRT or PBRT) seems necessary for local control also in case of negative resection margins.**

Response rate: 44/44

Rate of consensus: 87.8%

Outliers: 1

Statement approved? Yes

**S1.5 - Radiation therapy can be avoided in selected patients with small (<5 cm), low-grade, previously untreated tumors with negative microscopic margins.** (4–6)

Response rate: 46/47

Rate of consensus: 67.3%

Outliers: 1

Statement approved? No

**S1.5R - Even if a multidisciplinary discussion is always required, adjuvant radiation therapy can be avoided in selected cases of low-grade sarcomas not extending beyond the sinonasal compartment, surgically resected with negative microscopic margins (R0).**

Response rate: 44/44

Rate of consensus: 88.1%

Outliers: 1

Statement approved? Yes

**S1.6 - Tumor grade and histology represent the main prognosticators of sinonasal sarcoma.** (4–6)

Response rate: 45/47

Rate of consensus: 80%

Outliers: 4

Statement approved? No

**S1.6R - In case of sarcomas not presenting regional or distant metastasis, the tumor grade, stage, histology and surgical free resection margins represent the main prognosticators for sinonasal sarcomas.**

Response rate: 44/44

Rate of consensus: 92.5%

Outliers: 1

Statement approved? Yes

# **SESSION 2 - Angiosarcoma**

**S2.1 - In resectable cases, surgery with free margins represents the front-line treatment of choice.** (7)

Response rate: 44/47

Rate of consensus: 84%

Outliers: 4

Statement approved? No

**S2.1R - Given the challenges of sinonasal angiosarcoma resection and its systemic and often multifocal nature, systemic chemotherapy can be used in a neoadjuvant setting. However, in resectable cases, surgery with free margins (upfront or after induction chemotherapy) should be considered the curative treatment of choice.**

Response rate: 42/44

Rate of consensus: 92.5%

Outliers: 1

Statement approved? Yes

**S2.2 - Adjuvant radiotherapy (IMRT) is always indicated if no contraindications are present.** (8)

Response rate: 44/47

Rate of consensus: 77.2%

Outliers: 3

Statement approved? No

**S2.2R - Adjuvant radiotherapy (IMRT) is frequently indicated if no contraindications are present and/or in case of a low-grade tumor with free resection margin surgery.**

Response rate: 40/44

Rate of consensus: 86.8%

Outliers: 1

Statement approved? Yes

**S2.3 - In unresectable cases, exclusive radiotherapy (IMRT) is the treatment of choice since adjuvant chemotherapy does not usually influence the final outcome.** (8,9)

Response rate: 42/47

Rate of consensus: 50%

Outliers: 14

Statement approved? No

**S2.3R - In unresectable cases, a combination of radiotherapy (IMRT/IMPT) and chemotherapy is usually the treatment of choice.**

Response rate: 44/44

Rate of consensus: 100%

Outliers: 0

Statement approved? Yes

**S2.4 - In metastatic disease, exclusive chemotherapy is indicated, even if with scarce results.** (8,9)

Response rate: 40/47

Rate of consensus: 70%

Outliers: 8

Statement approved? No

**S2.4R - In metastatic disease, chemotherapy is the treatment of choice and can be associated, based on a multidisciplinary discussion, with:**

**- Surgery: in case of a single and easily resectable metastasis**

**- RT (IMRT): in case of an unresectable symptomatic single metastasis or oligometastatic disease (SBRT with a palliative intent).**

Response rate: 43/44

Rate of consensus: 97.5%

Outliers: 0

Statement approved? Yes

**S2.5 - Paclitaxel is the most used chemotherapeutic agent.** (9)

Response rate: 40/47

Rate of consensus: 70%

Outliers: 8

Statement approved? No

**S2.5R - Chemotherapy in a neoadjuvant/adjuvant setting is generally based on taxanes and/or anthracyclines, with and emerging role for antiangiogenics, notably tyrosine kinase inhibitors (TKIs), and immune checkpoint inhibitors (ICIs).**

Response rate: 38/44

Rate of consensus: 94.1%

Outliers: 0

Statement approved? Yes

# **SESSION 3 - Biphenotypic sinonasal sarcoma**

**S3.1 - In resectable cases, surgery with free margins represents the front-line treatment of choice.** (10)

Response rate: 46/47

Rate of consensus: 100%

Outliers: 0

Statement approved? Yes

**S3.2 - Adjuvant radiotherapy (IMRT or PBRT) is reserved to cases with R1-R2 margins.** (11)

Response rate: 46/47

Rate of consensus: 71.7%

Outliers: 3

Statement approved? No

**S3.2R - Adjuvant radiotherapy (IMRT or PBRT) is reserved for cases with R1-R2 margins, perineural or lymphovascular invasion and should be discussed in R0 high-stage disease.**

Response rate: 44/44

Rate of consensus: 92.8%

Outliers: 1

Statement approved? Yes

**S3.3 - In case of local (more frequent) or distant (rare) recurrence, surgery still represents the treatment of choice.** (11)

Response rate: 46/47

Rate of consensus: 80.4%

Outliers: 0

Statement approved? Yes

**S3.4 - Induction or adjuvant chemotherapy is not indicated a priori, considering its low-grade nature.** (12)

Response rate: 46/47

Rate of consensus: 91.3%

Outliers: 1

Statement approved? Yes

**S3.5 - In unresectable cases, radiotherapy (IMRT or PBRT) represents the treatment of choice.** (12,13)

Response rate: 46/47

Rate of consensus: 91.3%

Outliers: 0

Statement approved? Yes

# **SESSION 4 – Chondrosarcoma**

**S4.1 - Surgery with free margins represents the front-line treatment of choice.** (14–17)

Response rate: 46/47

Rate of consensus: 91.3%

Outliers: 0

Statement approved? Yes

**S4.2 - Considering its low-grade and indolent nature, in case of locally advanced tumor with vital structures involvement, the gross total resection vs maximal tumor resection (with macroscopic R2 margins) should be discussed taking into consideration predicted post-operative morbidities.** (18,19)

Response rate: 45/47

Rate of consensus: 82.2%

Outliers: 3

Statement approved? No

**S4.2R - In the case of locally advanced tumor with vital structures involvement, the R1/R2 surgery should be discussed taking into consideration expected post-operative morbidities.**

Response rate: 44/44

Rate of consensus: 88.1%

Outliers: 1

Statement approved? Yes

**S4.3 - Radiotherapy (IMRT or PBRT) is usually applied in case of recurrence or as adjuvant treatment in case of moderate-to-high-grade tumors and/or R1-R2 margins.** (20)

Response rate: 45/47

Rate of consensus: 75.5%

Outliers: 3

Statement approved? No

**S4.3R - After the primary surgical approach, adjuvant radiotherapy (IMRT or PBRT) should be multidisciplinary discussed and proposed to R1-R2 margins unresectable tumors or selected R0 margins tumors, with high-risk features.**

Response rate: 44/44

Rate of consensus: 85.7%

Outliers: 1

Statement approved? Yes

**S4.4 - Due to the better overall survival and local control, if radiotherapy is indicated, IMPT should be preferred to IMRT.** (21–23)

Response rate: 41/47

Rate of consensus: 73.1%

Outliers: 3

Statement approved? No

**S4.4R - IMPT provides better local control outcomes compared to IMRT, even if it lacks conclusive evidence.**

Response rate: 43/44

Rate of consensus: 87.5%

Outliers: 0

Statement approved? Yes

**S4.5 – Mesenchymal and dedifferentiated chondrosarcoma is thought to have increased chemosensitivity with a higher propensity to recur and metastasize. As part of multimodal therapy, chemotherapy is usually applied in neoadjuvant or adjuvant scenarios.** (20,24)

Response rate: 41/47

Rate of consensus: 92.5%

Outliers: 0

Statement approved? Yes

# **SESSION 5 – Ewing sarcoma**

**S5.1 - Neoadjuvant chemotherapy with 3-drugs or 4-drugs regimen is often indicated as front-line treatment in order to down-stage the primary tumor.** (25,26)

Response rate: 46/47

Rate of consensus: 91.3%

Outliers: 1

Statement approved? Yes

**S5.2 - Patients with stable or decrease tumor volume after primary neoadjuvant treatment should receive local control therapy based on:**

**- Surgical excision: if possible, based on tumor extension and patient comorbidities.**

**- Radiotherapy (IMRT or PBRT): if surgery is contraindicated.** (27–29)

Response rate: 46/47

Rate of consensus: 95.6%

Outliers: 1

Statement approved? Yes

**S5.3 - Adjuvant chemotherapy after surgical resection is generally recommended regardless of surgical margins.** (25,26)

Response rate: 46/47

Rate of consensus: 100%

Outliers: 0

Statement approved? Yes

**S5.4 - Adjuvant postoperative radiotherapy (IMRT or PBRT) is recommended in case of R1-R2 surgical margins.** (27–29)

Response rate: 45/47

Rate of consensus: 88.8%

Outliers: 1

Statement approved? Yes

**S5.5 - Patients with progressive disease after primary neoadjuvant chemotherapy should receive second lines chemotherapy associated with radiotherapy/surgery of the primary site for local control or palliation, based on a multidisciplinary discussion.** (30)

Response rate: 46/47

Rate of consensus: 84.7%

Outliers: 1

Statement approved? Yes

**S5.6 - Relapsed or refractory disease should be treated with chemotherapy with or without radiotherapy (IMRT or PBRT), based on tumor extension.** (30)

Response rate: 45/47

Rate of consensus: 93.3%

Outliers: 0

Statement approved? Yes

# **SESSION 6 – Leiomyosarcoma**

**S6.1 - In resectable cases, surgery with free margins represents the front-line treatment of choice.** (31,32)

Response rate: 44/47

Rate of consensus: 88.6%

Outliers: 1

Statement approved? Yes

**S6.2 - Adjuvant radiotherapy (IMRT) is primarily indicated in case of R1-R2 surgical margins.** (31,32)

Response rate: 44/47

Rate of consensus: 72.7%

Outliers: 6

Statement approved? No

**S6.2R - Adjuvant radiotherapy (IMRT) after the primary surgical approach should be multidisciplinary discussed and proposed to R1-R2 margins unresectable tumors or selected high-grade R0 margins tumors.**

Response rate: 43/44

Rate of consensus: 95.1%

Outliers: 0

Statement approved? Yes

**S6.3 - In unresectable cases, the application of neo-adjuvant chemotherapy followed by surgery or palliation radiotherapy (IMRT) should be discussed case-by-case.** (31,33)

Response rate: 43/47

Rate of consensus: 93%

Outliers: 0

Statement approved? Yes

**S6.4 - In metastatic cases, chemotherapy represents the treatment of choice, even if with scarce results.** (31,33)

Response rate: 43/47

Rate of consensus: 86%

Outliers: 3

Statement approved? No

**S6.4R - In metastatic cases, chemotherapy represents the treatment of choice; local therapies should be multidisciplinary discussed.**

Response rate: 44/44

Rate of consensus: 100%

Outliers: 0

Statement approved? Yes

# **SESSION 7 – Liposarcoma**

**S7.1 - In resectable cases, surgery with free margins represents the front-line treatment of choice.** (34,35)

Response rate: 45/47

Rate of consensus: 95.5%

Outliers: 0

Statement approved? Yes

**S7.2 - Adjuvant radiotherapy (IMRT or PBRT) is indicated in case of R1-R2 margins and/or high-grade tumor and/or large local extension.** (34,35)

Response rate: 45/47

Rate of consensus: 91.1%

Outliers: 0

Statement approved? Yes

**S7.3 - Neoadjuvant/adjuvant chemotherapy is not indicated a priori but can be discussed in case of high-grade liposarcomas and/or unresectable/metastatic cases.** (36)

Response rate: 45/47

Rate of consensus: 88.8%

Outliers: 0

Statement approved? Yes

# **SESSION 8 – Low-grade myofibroblastic sarcoma (Myofibrosarcoma)**

**S8.1 - In resectable cases, surgery with free margins represents the front-line treatment of choice.** (37,38)

Response rate: 44/47

Rate of consensus: 97.7%

Outliers: 0

Statement approved? Yes

**S8.2 - In unresectable cases, the application of neo-adjuvant chemotherapy followed by surgery or palliation radiotherapy (IMRT) should be discussed case-by-case.** (37,38)

Response rate: 43/47

Rate of consensus: 88.3%

Outliers: 0

Statement approved? Yes

**S8.3 - In metastatic cases (regional or distant), surgery associated with radiotherapy (IMRT) still represents the treatment of choice, with chemotherapy reserved to selected cases due to its general low response rate.** (39,40)

Response rate: 43/47

Rate of consensus: 83.7%

Outliers: 1

Statement approved? Yes

# **SESSION 9 – Malignant peripheral nerve sheat tumor**

**S9.1 - In resectable cases, surgery with free margins represents the front-line treatment of choice.** (41)

Response rate: 44/47

Rate of consensus: 88.6%

Outliers: 1

Statement approved? Yes

**S9.2 - Adjuvant radiotherapy (IMRT) is indicated in case of R1-R2 margins, high-grade tumor and/or large local extension.** (42)

Response rate: 44/47

Rate of consensus: 86.3%

Outliers: 3

Statement approved? No

**S9.2R - Adjuvant radiotherapy (IMRT) is generally indicated in R1-R2 margins, high-grade tumor and/or large local extensions. Its application in R0 cases should be discussed case-by-case.**

Response rate: 42/44

Rate of consensus: 87.5%

Outliers: 0

Statement approved? Yes

**S9.3 - Adjuvant chemotherapy after surgical excision is controversial and mainly indicated in high-grade tumor.** (43,44)

Response rate: 44/47

Rate of consensus: 90.9%

Outliers: 0

Statement approved? Yes

**S9.4 - In unresectable cases, palliative radiotherapy (IMRT) vs tumor maximal resection (with macroscopic R2 margins) followed by radiotherapy should be discussed case-by-case.** (43,44)

Response rate: 44/47

Rate of consensus: 84%

Outliers: 3

Statement approved? No

**S9.4R - In unresectable cases, palliative radiotherapy (IMRT/IMPT) vs palliative surgery followed by radiation therapy should be discussed case-by-case.**

Response rate: 42/44

Rate of consensus: 100%

Outliers: 0

Statement approved? Yes

**S9.5 - In metastatic cases (rare), chemotherapy represents the treatment of choice, primarily based on doxorubicin + ifosfamide regimen, even if with scarce results.** (43,44)

Response rate: 41/47

Rate of consensus: 85.3%

Outliers: 1

Statement approved? Yes

# **SESSION 10 – Osteosarcoma**

**S10.1 - In resectable cases, surgery with free margins represents the front-line treatment of choice.** (45)

Response rate: 46/47

Rate of consensus: 73.1%

Outliers: 9

Statement approved? No

**S10.1R - In resectable cases, surgery with free margins represents the front-line treatment of choice, with neo-adjuvant chemotherapy reserved for high-grade tumors.**

Response rate: 44/44

Rate of consensus: 95.2%

Outliers: 1

Statement approved? Yes

**S10.2 - Adjuvant radiotherapy (IMRT or PBRT) is primarily indicated in R1-R2 margins with IMPT being preferred in case of macroscopic (R2) residual disease.** (46,47)

Response rate: 43/47

Rate of consensus: 79.1%

Outliers: 2

Statement approved? No

**S10.2R - Adjuvant radiotherapy (IMRT or PBRT) is primarily indicated in R1-R2 margins and should be discussed in R0 margins cases, based on tumors grade.**

Response rate: 44/44

Rate of consensus: 95.2%

Outliers: 0

Statement approved? Yes

**S10.3 - Adjuvant chemotherapy is a possible solution, in addition to radiotherapy, in positive resection margin (R1-R2) and intermediate/high-grade histology.** (46,47)

Response rate: 45/47

Rate of consensus: 82.2%

Outliers: 3

Statement approved? No

**S10.3R - Adjuvant chemotherapy is a possible solution, in addition to radiotherapy, in high-grade tumors or in positive resection margins (R1-R2) tumors.**

Response rate: 43/44

Rate of consensus: 90.2%

Outliers: 0

Statement approved? Yes

**S10.4 - In unresectable tumors, IMPT is one of the primary options.** (45)

Response rate: 43/47

Rate of consensus: 81.3%

Outliers: 3

Statement approved? No

**S10.4R - In unresectable tumors, a combination of chemotherapy and radiotherapy (IMRT or IMPT) represents the treatment of choice.**

Response rate: 44/44

Rate of consensus: 95.2%

Outliers: 0

Statement approved? Yes

**S10.5 - Neoadjuvant chemotherapy is a possible solution in case of unresectable tumor and high-grade histology.** (48,49)

Response rate: 46/47

Rate of consensus: 91.3%

Outliers: 2

Statement approved? No

**S10.5R - Added to S10.4R**

**S10.6 - Chemotherapy is the treatment of choice of metastatic cases.** (48,49)

Response rate: 45/47

Rate of consensus: 95.5%

Outliers: 0

Statement approved? Yes

# **SESSION 11 – Rhabdomyosarcoma**

**S11.1 - Induction chemotherapy associated with curative chemo-radiotherapy (IMRT) represents the front-line treatment of choice.** (50)

Response rate: 46/47

Rate of consensus: 86.1%

Outliers: 2

Statement approved? No

**S11.1R - Induction chemotherapy is considered the upfront treatment of choice in most cases.**

Response rate: 44/44

Rate of consensus: 100%

Outliers: 0

Statement approved? Yes

**S11.2 - Surgery can be considered a solution instead of radiotherapy to address local disease control.** (51,52)

Response rate: 46/47

Rate of consensus: 63%

Outliers: 10

Statement approved? No

**S11.2R - A chemo-radiotherapy (IMRT/IMPT) approach is the treatment of choice after induction chemotherapy.**

Response rate: 44/44

Rate of consensus: 92.8%

Outliers: 1

Statement approved? Yes

**S11.3 - Surgery may be considered a routine component of multimodality treatment paradigms for RMS, particularly when a margin-negative resection is feasible with acceptable morbidity.** (53,54)

Response rate: 45/47

Rate of consensus: 82.2%

Outliers: 3

Statement approved? No

**S11.3R - In responders to chemotherapy with a localized lesion, surgical resection with free margin should be considered and evaluated case-by-case.**

Response rate: 44/44

Rate of consensus: 90.4%

Outliers: 1

Statement approved? Yes

**S11.4 - The most used chemotherapy regimens are vincristine, actinomycin D, and cyclophosphamide (VAC); vincristine, dactinomycin, and ifosfamide (VAI); vincristine, ifosfamide, and etoposide (VIE).**

Response rate: 44/47

Rate of consensus: 88.6%

Outliers: 1

Statement approved? Yes

**S11.5 - Intrathecal chemotherapy is no longer indicated in patients with evidence of tumoral cells inside the CSF.** (50)

Response rate: 35/47

Rate of consensus: 82.8%

Outliers: 1

Statement approved? Yes

**S11.6 - All patients with nonmetastatic relapse should receive a locoregional treatment primarily based on surgery.** (54,55)

Response rate: 45/47

Rate of consensus: 71.1%

Outliers: 4

Statement approved? No

**S11.6R - Most patients with nonmetastatic resectable relapse can receive a locoregional treatment based on surgery or RT (IMRT/IMPT), based on previous protocols, associated with adjuvant/neoadjuvant chemotherapy.**

Response rate: 44/44

Rate of consensus: 100%

Outliers: 0

Statement approved? Yes

**S11.7 - In case of unresectable recurrence, re-irradiation (IMRT) with second-line chemotherapy should be discussed.** (56)

Response rate: 45/47

Rate of consensus: 86.9%

Outliers: 0

Statement approved? Yes

**S11.8 - In case of metastatic pathology, chemotherapy is indicated based on previous treatments received.** (56)

Response rate: 45/47

Rate of consensus: 97.7%

Outliers: 0

Statement approved? Yes

**S11.9 - In pleomorphic rhabdomyosarcoma, due to its nature being more common to other soft tissue sarcomas, it is generally treated with upfront surgery +/− radiotherapy (IMRT) based on the margin status and histologic analysis.** (54,57)

Response rate: 43/47

Rate of consensus: 83.7%

Outliers: 1

Statement approved? Yes

# **SESSION 12 – Synovial sarcoma**

**S12.1 - In resectable cases, surgery with free margins represents the front-line treatment of choice.** (58)

Response rate: 44/47

Rate of consensus: 84%

Outliers: 5

Statement approved? No

**S12.1R - Surgery is the mainstay of treatment of sinonasal synovial sarcomas; however, chemotherapy can be used in neo-adjuvant setting, and should be discussed case-by-case.**

Response rate: 42/44

Rate of consensus: 95%

Outliers: 0

Statement approved? Yes

**S12.2 - Adjuvant radiotherapy (IMRT or PBRT) is reserved for cases with R1-R2 margins.** (59)

Response rate: 43/47

Rate of consensus: 47.7%

Outliers: 14

Statement approved? No

**S12.2R - Adjuvant radiotherapy (IMRT or PBRT) can improve local control both in R1-R2 margins and R0 cases with high-risk features, based on a multidisciplinary discussion.**

Response rate: 43/44

Rate of consensus: 97.5%

Outliers: 0

Statement approved? Yes

**S12.3 - Unresectable cases are generally treated with exclusive radiotherapy (IMRT or PBRT).** (60,61)

Response rate: 46/47

Rate of consensus: 51.1%

Outliers: 12

Statement approved? No

**S12.3R - Unresectable cases are generally treated with a combination of chemotherapy and radiotherapy (IMRT or PBRT).**

Response rate: 44/44

Rate of consensus: 97.6%

Outliers: 0

Statement approved? Yes

**S12.4 - Chemotherapy is usually reserved for metastatic cases, with anthracycline-based regimens being the treatment of choice.** (60,61)

Response rate: 41/47

Rate of consensus: 68.2%

Outliers: 10

Statement approved? No

**S12.4R - In metastatic cases, chemotherapy (anthracycline-based regimens) is the treatment of choice.**

Response rate: 41/44

Rate of consensus: 97.4%

Outliers: 0

Statement approved? Yes

**S12.5 - Pazopanib could be administrated in subsequent lines.** (62,63)

Response rate: 38/47

Rate of consensus: 89.4%

Outliers: 1

Statement approved? Yes

References

1. De Bree R, Van Der Waal I, De Bree E, René Leemans C. Management of adult soft tissue sarcomas of the head and neck. Oral Oncol [Internet]. 2010 Nov [cited 2025 Jun 2];46(11):786–90. Available from: https://pubmed.ncbi.nlm.nih.gov/20947413/

2. Peng KA, Grogan T, Wang MB. Head and neck sarcomas: Analysis of the SEER database. Otolaryngology - Head and Neck Surgery (United States) [Internet]. 2014 Oct 12 [cited 2025 Jun 2];151(4):627–33. Available from: https://pubmed.ncbi.nlm.nih.gov/25135525/

3. Kotecha S, Williams MA, White HB, Graystone J, Gibbons M, Cosker T. Head and neck sarcoma: three-year data from a tertiary referral centre. Ann R Coll Surg Engl [Internet]. 2021 Nov 1 [cited 2025 Jun 2];103(10):762–7. Available from: https://pubmed.ncbi.nlm.nih.gov/34448646/

4. Colville RJ, Charlton F, Kelly CG, Nicoll JJ, McLean NR. Multidisciplinary management of head and neck sarcomas. Head Neck [Internet]. 2005 Sep [cited 2025 Jun 2];27(9):814–24. Available from: https://pubmed.ncbi.nlm.nih.gov/16086411/

5. Fayda M, Aksu G, Yaman Agaoglu F, Karadeniz A, Darendeliler E, Altun M, et al. The role of surgery and radiotherapy in treatment of soft tissue sarcomas of the head and neck region: Review of 30 cases. Journal of Cranio-Maxillofacial Surgery [Internet]. 2009 Jan [cited 2025 Jun 2];37(1):42–8. Available from: https://pubmed.ncbi.nlm.nih.gov/18804382/

6. Tran LM, Mark R, Meier R, Calcaterra TC, Parker RG. Sarcomas of the head and neck. Prognostic factors and treatment strategies. Cancer [Internet]. 1992 [cited 2025 Jun 2];70(1):169–77. Available from: https://pubmed.ncbi.nlm.nih.gov/1606539/

7. Tomovic S, Kalyoussef E, Mirani NM, Baredes S, Eloy JA. Angiosarcoma arising from the frontal sinus. American Journal of Otolaryngology - Head and Neck Medicine and Surgery [Internet]. 2014 Nov 1 [cited 2025 Jun 8];35(6):806–9. Available from: https://pubmed.ncbi.nlm.nih.gov/25217371/

8. Young RJ, Brown NJ, Reed MW, Hughes D, Woll PJ. Angiosarcoma. Lancet Oncol [Internet]. 2010 Oct [cited 2025 Jun 8];11(10):983–91. Available from: https://pubmed.ncbi.nlm.nih.gov/20537949/

9. Van Glabbeke M, Van Oosterom AT, Oosterhuis JW, Mouridsen H, Crowther D, Somers R, et al. Prognostic factors for the outcome of chemotherapy in advanced soft tissue sarcoma: An analysis of 2,185 patients treated with anthracycline- containing first-line regimens - A European organization for research and treatment of cancer soft tissue and bone sarcoma group study. Journal of Clinical Oncology [Internet]. 1999 [cited 2025 Jun 8];17(1):150–7. Available from: https://pubmed.ncbi.nlm.nih.gov/10458228/

10. Oren N, Vaysberg A, Ginat DT. Updated WHO nomenclature of head and neck lesions and associated imaging findings. Insights Imaging [Internet]. 2019 Dec 1 [cited 2025 Jun 3];10(1). Available from: https://pubmed.ncbi.nlm.nih.gov/31312967/

11. Turri-Zanoni M, Dalfino G, Lechner M, Dallan I, Battaglia P, Facco C, et al. Biphenotypic sinonasal sarcoma: European multicentre case-series and systematic literature review. Acta Otorhinolaryngologica Italica [Internet]. 2022 Dec 1 [cited 2025 Jun 3];42(6):545–53. Available from: https://pubmed.ncbi.nlm.nih.gov/36654521/

12. Corvino S, Corazzelli G, Mariniello G, Iuliano A, Altieri R, Pontillo G, et al. Biphenotypic Sinonasal Sarcoma: Literature Review of a Peculiar Pathological Entity—The Neurosurgical Point of View. Cancers (Basel) [Internet]. 2024 Nov 1 [cited 2025 Jun 3];16(22). Available from: https://pubmed.ncbi.nlm.nih.gov/39594702/

13. Kominsky E, Boyke AE, Madani D, Kamat A, Schiff BA, Agarwal V. Biphenotypic Sinonasal Sarcoma: A Case Report and Review of Literature. Ear Nose Throat J [Internet]. 2023 Jun 1 [cited 2025 Jun 3];102(6):385–90. Available from: https://pubmed.ncbi.nlm.nih.gov/33813901/

14. Crockard HA, Cheeseman A, Steel T, Revesz T, Holton JL, Plowman N, et al. A multidisciplinary team approach to skull base chondrosarcomas. J Neurosurg [Internet]. 2001 [cited 2025 Jun 2];95(2):184–9. Available from: https://pubmed.ncbi.nlm.nih.gov/11780886/

15. Palmisciano P, Haider AS, Sabahi M, Nwagwu CD, Alamer O Bin, Scalia G, et al. Primary skull base chondrosarcomas: A systematic review. Cancers (Basel) [Internet]. 2021 Dec 1 [cited 2025 Jun 2];13(23). Available from: https://pubmed.ncbi.nlm.nih.gov/34885071/

16. Khan MN, Husain Q, Kanumuri V V., Boghani Z, Patel CR, Liu JK, et al. Management of sinonasal chondrosarcoma: A systematic review of 161 patients. Int Forum Allergy Rhinol [Internet]. 2013 Aug [cited 2025 Jun 2];3(8):670–7. Available from: https://pubmed.ncbi.nlm.nih.gov/23520030/

17. Kawaguchi N, Ahmed AR, Matsumoto S, Manabe J, Matsushita Y. The concept of curative margin in surgery for bone and soft tissue sarcoma. Clin Orthop Relat Res [Internet]. 2004 [cited 2024 Dec 13];419(419):165–72. Available from: https://pubmed.ncbi.nlm.nih.gov/15021149/

18. Eide JG, Kshirsagar RS, Harris JC, Civantos A, Brody RM, Lee JYK, et al. Multi-institutional review of sinonasal and skull base chondrosarcoma: 20-year experience. Head Neck [Internet]. 2022 Dec 1 [cited 2025 Jun 2];44(12):2686–95. Available from: https://pubmed.ncbi.nlm.nih.gov/36052545/

19. Samii A, Gerganov V, Herold C, Gharabaghi A, Hayashi N, Samii M. Surgical treatment of skull base chondrosarcomas. Neurosurg Rev [Internet]. 2009 Jan [cited 2025 Jun 2];32(1):67–75. Available from: https://pubmed.ncbi.nlm.nih.gov/18818961/

20. Raza SM, Gidley PW, Meis JM, Grosshans DR, Bell D, DeMonte F. Multimodality treatment of skull base chondrosarcomas: The role of histology specific treatment protocols. Neurosurgery [Internet]. 2017 Sep 1 [cited 2025 Jun 2];81(3):520–30. Available from: https://pubmed.ncbi.nlm.nih.gov/28368506/

21. Rimmer RA, Mace JC, Andersen PE, Cetas JS, Ciporen JN, Dogan A, et al. Determinants of survival in sinonasal and skull base chondrosarcoma: An analysis of the National Cancer Database. Int Forum Allergy Rhinol [Internet]. 2022 May 1 [cited 2025 Jun 2];12(5):699–713. Available from: https://pubmed.ncbi.nlm.nih.gov/34704402/

22. Hristov B, Shokek O, Frassica DA. The role of radiation treatment in the contemporary management of bone tumors. JNCCN Journal of the National Comprehensive Cancer Network [Internet]. 2007 [cited 2025 Jun 2];5(4):456–66. Available from: https://pubmed.ncbi.nlm.nih.gov/17442236/

23. Nakamura M, Mizumoto M, Saito T, Shimizu S, Li Y, Oshiro Y, et al. A systematic review and meta-analysis of radiotherapy and particle beam therapy for skull base chondrosarcoma: TRP-chondrosarcoma 2024. Front Oncol [Internet]. 2024 [cited 2025 Jun 2];14. Available from: https://pubmed.ncbi.nlm.nih.gov/38567162/

24. Colia V, Provenzano S, Hindi N, Casali PG, Stacchiotti S. Systemic therapy for selected skull base sarcomas: Chondrosarcoma, chordoma, giant cell tumour and solitary fibrous tumour/hemangiopericytoma. Reports of Practical Oncology and Radiotherapy [Internet]. 2016 Jul 1 [cited 2025 Jun 2];21(4):361–9. Available from: https://pubmed.ncbi.nlm.nih.gov/27330421/

25. Burgert EO. Multimodal therapy for the management of nonpelvic, localized Ewing’s sarcoma of bone: Intergroup study IESS-II. Journal of Clinical Oncology [Internet]. 1990 [cited 2025 Jun 4];8(9):1514–24. Available from: https://pubmed.ncbi.nlm.nih.gov/2099751/

26. Krasin MJ, Davidoff AM, Rodriguez-Galindo C, Billups CA, Fuller CE, Neel MD, et al. Definitive surgery and multiagent systemic therapy for patients with localized ewing sarcoma family of tumors: Local outcome and prognostic factors. Cancer [Internet]. 2005 Jul 15 [cited 2025 Jun 4];104(2):367–73. Available from: https://pubmed.ncbi.nlm.nih.gov/15948159/

27. Krasin MJ, Rodriguez-Galindo C, Billups CA, Davidoff AM, Neel MD, Merchant TE, et al. Definitive irradiation in multidisciplinary management of localized Ewing sarcoma family of tumors in pediatric patients: Outcome and prognostic factors. Int J Radiat Oncol Biol Phys [Internet]. 2004 Nov 1 [cited 2025 Jun 4];60(3):830–8. Available from: https://pubmed.ncbi.nlm.nih.gov/15465200/

28. Lepera D, Volpi L, Facco C, Turri-Zanoni M, Battaglia P, Bernasconi B, et al. Endoscopic treatment of ewing sarcoma of the sinonasal tract. Journal of Craniofacial Surgery [Internet]. 2016 [cited 2025 Jun 4];27(4):1001–6. Available from: https://pubmed.ncbi.nlm.nih.gov/27285893/

29. Lombardi D, Mattavelli D, Redaelli De Zinis LO, Accorona R, Morassi ML, Facchetti F, et al. Primary Ewing’s sarcoma of the sinonasal tract in adults: A challenging disease. Head Neck [Internet]. 2017 Mar 1 [cited 2025 Jun 4];39(3):E45–50. Available from: https://pubmed.ncbi.nlm.nih.gov/27898190/

30. Biermann JS, Chow W, Reed DR, Lucas D, Adkins DR, Agulnik M, et al. Bone cancer, version 2.2017 featured updates to the NCCN guidelines. JNCCN Journal of the National Comprehensive Cancer Network [Internet]. 2017 Feb 1 [cited 2025 Jun 4];15(2):155–67. Available from: https://pubmed.ncbi.nlm.nih.gov/28188186/

31. Saadoun R, Obermueller T, Franke M, Schell A, Mückner K, Riemann R. Leiomyosarcoma of the Nasal Cavity. Ear Nose Throat J [Internet]. 2022 Jun 1 [cited 2025 Jun 4];101(5):NP218–21. Available from: https://pubmed.ncbi.nlm.nih.gov/32951455/

32. Kwok MMK, Lee S, Hosking P. Leiomyosarcoma: A rare sinonasal malignancy. BMJ Case Rep [Internet]. 2018 [cited 2025 Jun 4];2018. Available from: https://pubmed.ncbi.nlm.nih.gov/29588301/

33. Ulrich CT, Feiz-Erfan I, Spetzler RF, Isaacs JD, Hott JS, Nakaji P, et al. Sinonasal leiomyosarcoma: Review of literature and case report. Laryngoscope [Internet]. 2005 Dec [cited 2025 Jun 4];115(12):2242–8. Available from: https://pubmed.ncbi.nlm.nih.gov/16369174/

34. Gerry D, Fox NF, Spruill LS, Lentsch EJ. Liposarcoma of the head and neck: Analysis of 318 cases with comparison to non-head and neck sites. Head Neck [Internet]. 2014 Mar [cited 2025 Jun 8];36(3):393–400. Available from: https://pubmed.ncbi.nlm.nih.gov/23728920/

35. Davis EC, Ballo MT, Luna MA, Patel SR, Roberts DB, Nong X, et al. LIPOSARCOMA of the head and neck: the university of texas M. D. anderson cancer center experience. Head Neck [Internet]. 2009 Jan [cited 2025 Jun 8];31(1):28–36. Available from: https://pubmed.ncbi.nlm.nih.gov/18767171/

36. Barisella M, Giannini L, Piazza C. From head and neck lipoma to liposarcoma: A wide spectrum of differential diagnoses and their therapeutic implications. Curr Opin Otolaryngol Head Neck Surg [Internet]. 2020 Apr 1 [cited 2025 Jun 8];28(2):136–43. Available from: https://pubmed.ncbi.nlm.nih.gov/32011399/

37. Bughrara MS, Almsaddi T, John J, Prentice B, Johnson J, Henriquez O, et al. Fibrosarcomas of the Paranasal Sinuses: A Systematic Review. Cureus [Internet]. 2022 Aug 11 [cited 2025 Jun 4];14(8). Available from: https://pubmed.ncbi.nlm.nih.gov/36110445/

38. Fu Y ‐S, Perzin KH. Nonepithelial tumors of the nasal cavity, paranasal sinuses, and nasopharynx. A clinicopathologic study VI. Fibrous tissue tumors (fibroma, fibromatosis, fibrosarcoma). Cancer [Internet]. 1976 [cited 2025 Jun 4];37(6):2912–28. Available from: https://pubmed.ncbi.nlm.nih.gov/949712/

39. Lartigau E, Lusinchi A, Schwaab G. Sarcomas of nasal cavity and paranasal sinuses: Chondrosarcoma, osteosarcoma and fibrosarcoma. J Laryngol Otol [Internet]. 1994 [cited 2025 Jun 4];108(11):947–53. Available from: https://pubmed.ncbi.nlm.nih.gov/7829947/

40. Patel TD, Carniol ET, Vázquez A, Baredes S, Liu JK, Eloy JA. Sinonasal fibrosarcoma: Analysis of the Surveillance, Epidemiology, and End Results database. Int Forum Allergy Rhinol [Internet]. 2016 Feb 1 [cited 2025 Jun 4];6(2):201–5. Available from: https://pubmed.ncbi.nlm.nih.gov/26370489/

41. Minovi A, Basten O, Hunter B, Draf W, Bockmühl U. Malignant peripheral nerve sheath tumors of the head and neck: Management of 10 cases and literature review. Head Neck [Internet]. 2007 May [cited 2025 Jun 11];29(5):439–45. Available from: https://pubmed.ncbi.nlm.nih.gov/17163467/

42. Carli M, Ferrari A, Mattke A, Zanetti I, Casanova M, Bisogno G, et al. Pediatric malignant peripheral nerve sheath tumor: The Italian and German Soft Tissue Sarcoma Cooperative Group. Journal of Clinical Oncology [Internet]. 2005 [cited 2025 Jun 17];23(33):8422–30. Available from: https://pubmed.ncbi.nlm.nih.gov/16293873/

43. DeCou JM, Rao BN, Parham DM, Lobe TE, Bowman L, Pappo AS, et al. Malignant peripheral nerve sheath tumors: The St. Jude Children’s Research Hospital experience. Ann Surg Oncol [Internet]. 1995 Nov [cited 2025 Jun 17];2(6):524–9. Available from: https://pubmed.ncbi.nlm.nih.gov/8591083/

44. Santoro A, Tursz T, Mouridsen H, Verweij J, Steward W, Somers R, et al. Doxorubicin versus CYVADIC versus doxorubicin plus ifosfamide in first-line treatment of advanced soft tissue sarcomas: A randomized study of the European Organization for Research and Treatment of Cancer Soft Tissue and Bone Sarcoma Group. Journal of Clinical Oncology [Internet]. 1995 [cited 2025 Jun 17];13(7):1537–45. Available from: https://pubmed.ncbi.nlm.nih.gov/7602342/

45. Low CM, Gruszczynski NR, Moore EJ, Price DL, Janus JR, Kasperbauer JL, et al. Sinonasal Osteosarcoma: Report of 14 New Cases and Systematic Review of the Literature. J Neurol Surg B Skull Base [Internet]. 2020 Jul 1 [cited 2025 Jun 13];82(Suppl 3):e138. Available from: https://pmc.ncbi.nlm.nih.gov/articles/PMC8289535/

46. Smith RB, Apostolakis LW, Karnell LH, Koch BB, Robinson RA, Zhen W, et al. National cancer data base report on osteosarcoma of the head and neck. Cancer [Internet]. 2003 Oct 15 [cited 2025 Jun 13];98(8):1670–80. Available from: https://pubmed.ncbi.nlm.nih.gov/14534884/

47. Oda D, Bavisotto LM, Schmidt RA, McNutt M, Bruckner JD, Conrad EU, et al. Head and neck osteosarcoma at the University of Washington. Head Neck [Internet]. 1997 [cited 2025 Jun 13];19(6):513–23. Available from: https://pubmed.ncbi.nlm.nih.gov/9278760/

48. Boon E, van der Graaf WTA, Gelderblom H, Tesselaar MET, van Es RJJ, Oosting SF, et al. Impact of chemotherapy on the outcome of osteosarcoma of the head and neck in adults. Head Neck [Internet]. 2016 Jan 1 [cited 2025 Jun 13];39(1):140. Available from: https://pmc.ncbi.nlm.nih.gov/articles/PMC5215442/

49. Liang L, Zhang T, You Y, He Q, Fan Y, Liao G. An individual patient data meta-analysis on the effect of chemotherapy on survival in patients with craniofacial osteosarcoma. Head Neck [Internet]. 2019 Jun 1 [cited 2025 Jun 13];41(6):2016–23. Available from: https://pubmed.ncbi.nlm.nih.gov/30706580/

50. Beverly Raney R, Maurer HM, Anderson JR, Andrassy RJ, Donaldson SS, Qualman SJ, et al. The Intergroup Rhabdomyosarcoma Study Group (IRSG): Major lessons from the IRS-I through IRS-IV studies as background for the current IRS-V treatment protocols. Sarcoma [Internet]. 2001 [cited 2025 Jun 7];5(1):9–15. Available from: https://pubmed.ncbi.nlm.nih.gov/18521303/

51. Casey DL, Wolden SL. Rhabdomyosarcoma of the Head and Neck: A Multimodal Approach. J Neurol Surg B Skull Base [Internet]. 2018 Feb 1 [cited 2025 Jun 7];79(1):58–64. Available from: https://pubmed.ncbi.nlm.nih.gov/29404242/

52. Stevens MCG, Rey A, Bouvet N, Ellershaw C, Flamant F, Habrand JL, et al. Treatment of nonmetastatic rhabdomyosarcoma in childhood and adolescence: Third study of the International Society of Paediatric Oncology-SIOP malignant mesenchymal tumor 89. Journal of Clinical Oncology [Internet]. 2005 Apr 20 [cited 2025 Jun 7];23(12):2618–28. Available from: https://pubmed.ncbi.nlm.nih.gov/15728225/

53. Rodeberg DA, Wharam MD, Lyden ER, Stoner JA, Brown K, Wolden SL, et al. Delayed primary excision with subsequent modification of radiotherapy dose for intermediate-risk rhabdomyosarcoma: A report from the Children’s Oncology Group Soft Tissue Sarcoma Committee. Int J Cancer [Internet]. 2015 Jul 1 [cited 2025 Jun 7];137(1):204–11. Available from: https://pubmed.ncbi.nlm.nih.gov/25418440/

54. Kana LA, Smith JD, Bellile EL, Chugh R, McKean EL. Surgical Management of Rhabdomyosarcoma of the Nasal Cavity and Paranasal Sinuses: Analysis of Operative Indications, Settings, and Outcomes. J Neurol Surg B Skull Base [Internet]. 2021 Apr 19 [cited 2025 Jun 7];83(4):350–8. Available from: https://pubmed.ncbi.nlm.nih.gov/35903658/

55. Paulino AC, Bauman N, Simon JH, Nguyen TX, Ritchie JM, Tannous R. Local control of parameningeal rhabdomyosarcoma: Outcomes in non-complete responders to chemoradiation. Med Pediatr Oncol [Internet]. 2003 Aug 1 [cited 2025 Jun 7];41(2):118–22. Available from: https://pubmed.ncbi.nlm.nih.gov/12825215/

56. Sevrin F, Bogart E, Orbach D, Le Deley MC, Berlanga P, Bernier V, et al. Therapy and Outcomes of Patients with Relapsed Nonmetastatic Rhabdomyosarcoma: A Report from the French Society of Pediatric Oncology Malignant Mesenchymal Tumor Committee. Cancer Med [Internet]. 2024 Dec 1 [cited 2025 Jun 7];13(23). Available from: https://pubmed.ncbi.nlm.nih.gov/39610307/

57. Hahn E, Barot S, O’Sullivan B, Huang SH, Gupta A, Hosni A, et al. Adult Head and Neck Rhabdomyosarcoma: Management, Outcomes, and the Effect of Intensity Modulated Radiation Therapy on Locoregional Control. Adv Radiat Oncol [Internet]. 2022 Nov 1 [cited 2025 Jun 7];7(6). Available from: https://pubmed.ncbi.nlm.nih.gov/36420200/

58. Saito S, Ozawa H, Ikari Y, Nakahara N, Ito F, Sekimizu M, et al. Synovial sarcoma of the maxillary sinus: An extremely rare case with excellent response to chemotherapy. Onco Targets Ther [Internet]. 2018 Jan 23 [cited 2025 Jun 11];11:483–8. Available from: https://pubmed.ncbi.nlm.nih.gov/29416348/

59. Sharabati HH, Innab LR, Hussein SS, Salman AA, Naser AM, Bakri IA. Synovial sarcoma of the ethmoid sinus with extension to the cavernous sinus: a case report and literature review. J Surg Case Rep [Internet]. 2024 Oct 1 [cited 2025 Jun 11];2024(10). Available from: https://pubmed.ncbi.nlm.nih.gov/39364429/

60. Rosen G, Forscher C, Lowenbraun S, Eilber F, Eckardt J, Holmes C, et al. Synovial sarcoma. Uniform response of metastases to high dose ifosfamide. Cancer [Internet]. 1994 [cited 2025 Jun 11];73(10):2506–11. Available from: https://pubmed.ncbi.nlm.nih.gov/8174046/

61. Kampe CE, Rosen G, Eilber F, Eckardt J, Lowenbraun S, Foster J, et al. Synovial sarcoma. A study of intensive chemotherapy in 14 patients with localized disease. Cancer [Internet]. 1993 [cited 2025 Jun 11];72(7):2161–9. Available from: https://pubmed.ncbi.nlm.nih.gov/8397060/

62. Pokras S, Tseng WY, Espirito JL, Beeks A, Culver K, Nadler E. Treatment patterns and outcomes in metastatic synovial sarcoma: a real-world study in the US oncology network. Future Oncology [Internet]. 2022 [cited 2025 Jun 11];18(32):3637–50. Available from: https://pubmed.ncbi.nlm.nih.gov/36018238/

63. Desar IME, Fleuren EDG, van der Graaf WTA. Systemic Treatment for Adults with Synovial Sarcoma. Curr Treat Options Oncol [Internet]. 2018 Feb 1 [cited 2025 Jun 11];19(2). Available from: https://pubmed.ncbi.nlm.nih.gov/29516254/
